# Group-based physical activity trajectories in children transitioning from elementary to high school

**DOI:** 10.1186/s12889-019-6630-7

**Published:** 2019-03-18

**Authors:** Russell R. Pate, Michaela A. Schenkelberg, Marsha Dowda, Kerry L. McIver

**Affiliations:** Department of Exercise Science, Public Health Research Center, University of South Carolina, 921 Assembly Street, Suite 212, Columbia, SC 29201 USA

**Keywords:** Physical activity, Children, Age-related trajectories

## Abstract

**Background:**

Physical activity has been observed repeatedly to decline as children transition into adolescence; however, few studies have explored the possibility that sub-groups of children experience unique patterns of change during this transition. The purpose of this longitudinal study was to examine the physical activity trajectories in clusters of youth transitioning from 5th to 11th grade.

**Methods:**

Participants (*n* = 652) were recruited as 5th graders (ages 10–12 years) from elementary schools (*n* = 21) in two school districts. Demographic, anthropometric, and physical activity data were collected once per year when children were in 5th, 6th, 7th, 9th, and 11th grades. Children wore accelerometers for 7 consecutive days. Group-based trajectory modeling statistical techniques were applied to identify patterns of physical activity trajectories. Posterior probabilities confirmed participants’ membership in their respective group.

**Results:**

Three distinct physical activity trajectories were identified. Group 1 (*n* = 27) remained highly active over time, and physical activity increased from ages 14 to 16 years. Group 2 (*n* = 365) was active at baseline, but activity declined and remained low as group members aged. Group 3 (*n* = 260) had the lowest levels of physical activity at all ages, and activity declined from ages 10 to 16 years.

**Conclusions:**

While most children experienced a decline in physical activity as they transitioned into high school, some remained highly active and increased their level of physical activity. Future studies should test physical activity interventions for youth that are tailored for age-related trajectory groups.

## Background

An important public health priority is to increase the amount of time children (6–12 years) and adolescents (13–18 years) spend in physical activity. Empirical evidence consistently supports the relationships between physical activity and positive physical, psychological, and cognitive outcomes [1–3]. Participation in physical activity during childhood tracks over time and may influence health and development as children transition into adolescence and adulthood [4]. Therefore, public health guidelines recommend that children and adolescents participate in at least 60 min of moderate-to-vigorous physical activity each day [1]. However, most children and youth do not meet these guidelines [5]. Further, evidence suggests that girls are considerably less active than boys [5–8] and that physical activity declines markedly as children age, especially during adolescence [5, 6, 9].

Longitudinal studies have reported similar rates of decline in physical activity among boys and girls throughout adolescence [10, 11]. Nader and colleagues used accelerometers to assess the physical activity levels of a geographically diverse sample of 9-year-old children from the Study of Early Child Care and Youth Development until they were 15 years old (*n* = 1032) [11]. Overall, boys were significantly more active than girls, and physical activity declined drastically as children aged. At age 9 years, 90% of children were achieving the recommended 60 min of moderate-to-vigorous physical activity; however, by age 15 years, only 17% achieved the recommended level on weekend days and 31% on weekdays [11]. The most recent review of longitudinal studies estimated that physical activity declines, on average, 7% each year during adolescence [10]. This estimate, however, was based primarily on subjective measures of physical activity, because only 5 of the 26 studies reviewed utilized objective measures (pedometers, *n* = 3; accelerometers, *n* = 2). Further, most studies utilized only two time-points for data analyses, which presents difficulties in estimating the timing and magnitude of the decline in physical activity. Recent longitudinal studies applied group-based trajectory modeling as an analytical strategy to further examine the timing and trajectories of physical activity declines from childhood to adolescence [12–14]. Group-based trajectory modeling is a statistical approach designed to identify subgroups of individuals who follow a similar pattern of behavior over time. These studies revealed that children may follow one of several distinct physical activity trajectories over time (e.g. declining or increasing).

Considering the limitations of the longitudinal studies reported in Dumith et al., [10] it is prudent for researchers to reexamine the common view that physical activity declines in adolescence. Contemporary studies not only suggest that the declines may begin before adolescence, but also indicate that children may follow different, distinct physical activity trajectories over time. Such information can have important public health implications. However, to our knowledge, only three studies have explored these trajectories using group-based trajectory modeling: one among a homogenous sample (94% White) of American children [12], another among girls (54% Black) with self-reported physical activity [12–14], and the most recent among a sample of English children [13]. The present study aims to apply group-based trajectory modeling to explore the physical activity trajectories of a diverse sample of American youth as they transition from 5th (ages 10–12 years) to 11th grade (ages 16–18 years).

## Methods

### Participants and setting

Fifth grade participants were enrolled in the Transitions and Activity Changes in Kids (TRACK) Study, a multilevel longitudinal study, and included students from 21 elementary schools in two school districts in South Carolina. All 7 schools in one district, and 14 of the 17 schools in the second district agreed to participate. Details regarding the protocol for the parent study and student recruitment procedures are reported elsewhere [15]. Prior to data collection, parent or guardian written consent and child assent were obtained. Each data collection period consisted of two visits to the school and occurred once per year. During the initial visit, participating students received an accelerometer and completed anthropometric measurements and a student questionnaire. Students returned the accelerometer during the second visit. Parents completed a questionnaire providing demographic information about the child and family members. Parental education level was taken as a surrogate measure of family socio-economic status. Parents selected one of six optional education levels, ranging from “attends or has attended high school” to “completed graduate school.” For this study two categories were created, corresponding to high school graduation or less, and attendance at college/technical school or more. The Institutional Review Board at the University of South Carolina approved all study protocols.

Students self-reported their age, sex, race (American Indian or Alaskan Native, Asian, Black or African American, Native Hawaiian or Pacific Islander, White, or Other) and ethnicity (Hispanic or Latino). Race and ethnicity variables were combined and collapsed into the following categories: Non-Hispanic White, Non-Hispanic Black, Hispanic, and Other, which included multi-racial. A total of 1083 children (501 boys, 579 girls) were recruited into the TRACK study in 2010 (35.1% Black, 36.4% White, 11.2% Hispanic and 17.3% Other races/ethnicities). Mean age and body mass index (BMI) were 10.6 (± 0.6) years and 21.2 (± 4.9) kg/m^2^, respectively. In the 5th grade, a total of 992 children (92%) provided baseline accelerometer data for assessment of physical activity. All measures were repeated in 6th, 7th, 9th, and 11th grades. Students were excluded from the analyses if data were missing for parent education, an indicator of socioeconomic status (*n* = 47). There were no differences by race or gender between the 945 with and the 47 without parent education. Only those who completed at least three out of the five accelerometry assessments were included in the current study. There were no differences by gender, race, or parent education between students included (*n* = 652 and excluded (*n* = 292) from the study.

### Measures

#### Physical activity

Physical activity (light, moderate, and vigorous) was objectively measured using accelerometers (ActiGraph GT1M and GT3X models, Pensacola, FL). Each child wore an accelerometer during waking hours for 7 consecutive days, except while bathing or swimming. Accelerometer data were collected and stored in 60-s epochs. Any period of 60 or more minutes of consecutive zeroes was considered non-wear time and was set to missing. A threshold of 100 counts per minute (cpm) differentiated sedentary (≤100 cpm) from physical activity (> 100 cpm). Children included in the analyses had worn the accelerometer for at least 8 hours on two or more days at three or more time points. Among those children, 77% of potential physical activity values were available for Mondays through Saturdays. Missing values were estimated by multiple imputation using Proc MI in SAS (Version 9.3, SAS Institute). Total physical activity time (minutes) was divided by monitor wear time (hours) to express physical activity as minutes per hour.

#### Anthropometry

All anthropometric measurements took place at the school in small groups settings (≤24 students). Students were instructed to remove heavy clothing and shoes, and trained staff conducted two trials of height and weight assessments. Students’ standing and seated heights were measured to the nearest 0.1 cm using a portable stadiometer (Seca, Hamburg, Germany). Weight was measured to the nearest 0.1 kg using an electronic scale (Model 770; Seca, Hamburg, Germany). The average of two measurements was used for both height and weight, and BMI was calculated using the standard equation (kg/m^2^). Maturity offset, a noninvasive method to estimate peak-height velocity, was calculated using anthropometric variables and gender-specific equations [16, 17]. A negative maturity offset represents the number of years the child is from reaching peak height velocity, while a positive maturity offset represents the number of years a child is beyond peak height velocity.

### Statistical analysis

To identify patterns of trajectories of physical activity, group-based trajectory analysis with PROC TRAJ [18] in SAS (version 9.4) was conducted using the CNORM distribution for continuous data. Both linear and quadratic trajectories for 1, 2, 3 and 4 groups were tested. The final number of groups was determined by using Bayesian Information Criterion (BIC), the proportion of participants in each group, and the change in BIC between models (estimate of logged Bayes factor-2∆BIC) [18]. A 10-fold difference in Bayes factor is considered a meaningful difference. As confirmation for the number of groups chosen, posterior probabilities and odds of correct classification (OCC) were calculated [19, 20]. Posterior probability values greater than 0.70 indicate that the trajectory includes subjects with similar patterns of change, and an OCC of 5 or more is generally recommended for all groups [19, 20]. After the number of groups was determined, models were rerun eliminating non-significant quadratic terms. Analyses were conducted to determine if there were differences in demographic variables among the groups.

## Results

Of the 652 participants included in the analyses, 46% were boys, 36% were Black, 38% White, 9% Hispanic, and 17% were of other race/ethnic groups (including multi-racial). Fifty-eight percent of children had one or more parents with greater than a high school education (Table 1). Mothers were the primary respondent (87.3%) for the parent survey. Data were collected 5 times from 5th through 11th grade (ages 10–16 years). Students had 3, 4, or 5 valid accelerometer assessments across the span of the study (62, 19, and 19%, respectively). The trajectory of physical activity (min/hr) declined from age 10 to 16 years.

**Table 1:** Characteristics of study participants

Characteristic	Total *n* = 652	Males *n* = 297	Females *n* = 355	*p*-value*
Age (Mean ± SD), years	10.6 (0.5)	10.6 (0.5)	10.5 (0.5)	.82
Race (%)				
Black	36.2%	39.1%	33.8%	.53
White	37.9%	35.7%	39.7%	
Hispanic	9.2%	9.4%	9.0%	
Other	16.7%	15.8%	17.5%	
Parent Education, % Beyond high school	57.8%	56.1%	59.9%	.32
Maturity index at baseline, (Mean ± SD)	−1.63 (1.11)	−2.61 (0.6)	−0.81 (0.7)	<.001
BMI at baseline, (Mean ± SD)	21.2 (5.0)	20.7 (4.9)	21.6 (5.0)	.03
Physical activity Min/hr., (Mean ± SD)				
5th, n = 652	28.3 (4.5)	29.3 (4.7)	27.5 (4.2)	<.001
6th, *n* = 617	24.3 (4.5)	25.9 (4.2)	23.0 (4.3)	<.001
7th, *n* = 616	22.7 (4.6)	24.2 (4.5)	21.4 (4.4)	<.001
9th, *n* = 230	19.4 (5.5)	21.3 (6.3)	19.9 (4.2)	<.001
11th,*n* = 207	18.9 (6.5)	21.3 (7.1)	17.2 (5.4)	<.001

*p-value for difference between males and females

**Fig. 1:**
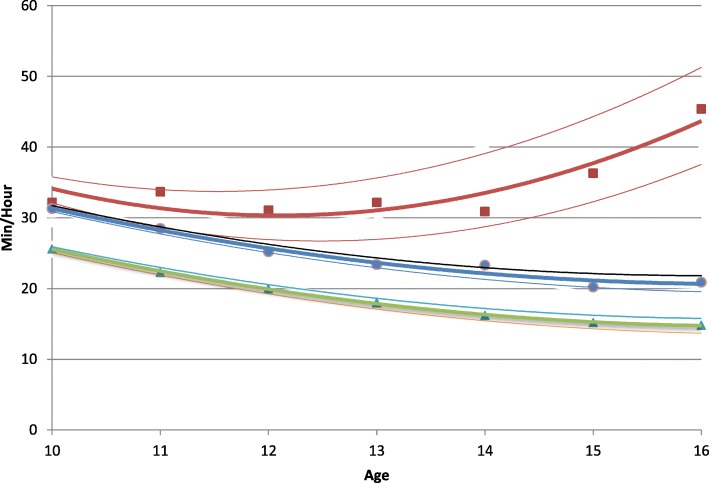
Physical activity trajectory groups in children ages 10–16 years.  Group 1.  Group 2.  Group 3

Three physical activity trajectories were identified (Table 2) using BIC, 2∆BIC, and consideration of group sizes. These three distinct trajectories were supported by posterior probabilities for each group of greater than .80 and odds of correct classifications (OCC) > 5. Posterior probability was 0.85 (OCC = 110.0), 0.87 (OCC = 5.5), and 0.87 (OCC = 9.8) for Group 1, 2, and 3, respectively. Physical activity of participants in Group 1 (*n* = 27) remained high over time and increased from ages 14 to 16 years (see Fig. 1). Baseline physical activity of participants in Group 2 (*n* = 365) was similar to Group 1; however, physical activity declined and remained consistently lower as children aged. Participants in Group 3 (*n* = 260) had the lowest physical activity at all ages and declined from 10 to 16 years of age.

**Table 2:** Determining number of groups for total participants

	1 Group	2 Groups			1 vs 2*	3 Groups				2 vs 3*	4 Groups					3 vs 4*
	BIC	BIC	% per group		2∆BIC	BIC	% per group			2∆BIC	BIC	% per group				2∆BIC
Total PA	− 6460	− 6320	53.8	46.2	278.2	− 6259	4.9	40.5	54.7	124.2	− 6234	6.2	2.3	35.8	55.6	49.6

Notes

1. BIC = Bayesian information criterion, smaller is better

2. * Comparision between groups; Interpretation of 2∆BIC = estimate of 2log_e_ (B_10_) [18]. Evidence against simpler model: 0–2 = not worth mentioning; 2–6 = Positive; 6–10 = Strong; > 10 = Very strong

Characteristics of the three trajectory groups are presented in Table 3. Group 1, which was consistently most physically active, was predominately male (77.8%), and in the 5th grade it had the lowest mean BMI and was the least physically mature of the three groups. Group 3 was consistently the least physically active, and it was predominately female (75.4%), was the most physically mature, and had the highest BMI. Group 2, which manifested an activity level between the other two groups, was more balanced in sex distribution (58.1% male) and was midway between the other groups in BMI and maturity in 5th grade.

**Table 3:** Characteristics of participants by physical activity trajectory groups

	Group 1	Group 2	Group 3	*p*-value
	*N* = 27	*N* = 365	*N* = 260	
Males, %	77.8%	58.1%	24.6%	<.001
Race				.26
Black, %	51.9%	41.5%	30.8%	
White, %	29.6%	35.9%	41.5%	
Hispanic, %	7.4%	8.5%	10.4%	
Other, %	11.1%	8.5%	17.3%	
Parent > high school Education, %	48.2%	55.1%	62.7%	.10
Maturity offset, 5th grade, mean (SD)	−2.3 (1.0)	−1.8 (1.1)	−1.3 (1.0)	<.001
BMI, 5th grade	19.3 (5.3)	20.9 (4.7)	21.7 (5.3)	.02
BMI z-score, 5th grade	0.4 (1.0)	0.86 (1.0)	0.99 (1.1)	.01

## Discussion

Previous studies have shown that physical activity tends to decrease with increasing age during childhood and adolescence [5, 6, 9, 10]. However, many of those studies used cross-sectional designs that may produce findings that do not accurately reflect trends that would be evident with prospective, longitudinal study designs [10]. Also, few of the previous longitudinal studies of physical activity have considered the possibility that sub-groups of youth may show different patterns of change as they transition from childhood to adolescence [10]. The major finding of the present study was that three distinct patterns of change in physical activity emerged when objectively measured physical activity was observed in a diverse sample of youth as they transitioned from 5th to 11th grade. Two of the groups manifested similar curvilinear decreases in physical activity, with plateaus developing at age 15 years. For these groups, the per year decrease in physical activity was approximately 2 min per hour, or 20–24 min per day. The cumulative five-year decline between ages 10 and 15 was 100–120 min per day. The two groups differed in physical activity level, and that difference was consistent across the period observed. A third group showed a pattern that was very distinct from the other two groups. Youth in this group showed no change in physical activity between ages 10 and 14 years, and then showed increased physical activity between ages 14 and 16 years. Although the number of participants in the third group was very small, the distinct pattern observed in that group demonstrates that some youth maintain, or even increase, their physical activity levels during a developmental period when most decrease participation in physical activity. This is important because reducing the age-related decline in physical activity is a key public health objective [1].

Other studies have noted similar physical activity trajectories as children transition to adolescence. Kwon and colleagues examined moderate-to-vigorous physical activity levels of a cohort of participants (94% White) in the Iowa Bone Development Study [12]. Physical activity of 537 children was assessed with accelerometry from ages 5 years to 20 years, and group-based trajectory analyses were applied. Four distinct physical activity trajectories were identified, and most participants (~ 70%) declined in moderate-to-vigorous activity over time. However, one group remained consistently active from childhood through adolescence [12]. A similar longitudinal study of children in the UK (*n* = 545) identified three different physical activity trajectories for boys and one trajectory for girls and found that all groups declined in overall physical activity from ages 7 years to 15 years [13]. The present study utilized similar group-based trajectory analysis methodologies as Kwon et al. [12] and Farooq et al. [13] in a racially and ethnically diverse sample of American children as they transitioned to adolescence. Consistent with both of the other studies, the present study found that, within a diverse sample of children, distinct patterns of change in physical activity were evident.

The association between biological maturation and physical activity is not well understood, but some have argued that they may be closely related [21, 22]. It may be that maturation directly influences physical activity levels. For example, in a study of 268 adolescent girls (age 10–12 years), girls who matured early (as determined by peak height velocity) tended to be heavier and less physically active than average or late maturing girls [22]. In contrast, it has been reported that early maturing boys are more physically active than later maturers [23]. Or it may be that maturation indirectly influences physical activity through psychosocial factors and social norms [17]. In the present study the three trajectory groups differed significantly in maturity offset at baseline (*p* < .001). The most active trajectory group (Group 1) was least physically mature as 5th graders, and the least active group (Group 3) was the most mature. This observation is at least partially explained by the uneven distribution of boys and girls across the three trajectory groups. Boys, who were less mature and more active at baseline, were disproportionally represented in Group 1. In contrast, girls, who were more mature and less active at baseline, were disproportionately represented in Group 3. Further, our findings indicate that maturity at baseline is predictive of future physical activity levels, since students who were least mature and most active in 5th grade tended to remain most active during the transition to 11th grade. Future studies should be designed to determine if the relationship between maturity and physical activity is causal or explained by other factors that may be associated with development during the peripubertal period.

In the present study we opted to conduct a group-based trajectory analysis in a diverse sample of children, including both girls and boys. We found that both sexes, though unevenly distributed, were significantly represented in all three trajectory groups. Girls were most highly represented in Group 3, the least active of the three groups, but girls also accounted for nearly a quarter of those in Group 1, the group that was most highly active and remained so across the period of observation. It is well documented that girls, on average, tend to be less physically active than boys [10, 24], and that was true in the present study (see Table 1). It is also clear that the sexes tend to differ in some factors that exert important influences on physical activity behavior [25, 26]. However, it is important to note that physical activity levels and factors influencing physical activity are highly variable in both sexes [25, 26]. Our findings demonstrate that, when age-related change in physical activity is examined longitudinally, patterns are observed that apply to groups of children, each of which are diverse in terms of gender as well as other demographic characteristics. This observation suggests that interventions to promote increased physical activity should consider children’s physical activity behavior patterns and should not be based primarily on demographic factors such as sex.

Several limitations of the present study should be considered. First, not all participants perfectly followed the patterns of their respective physical activity trajectory group. However, the posterior probabilities were well above the minimum value of 0.70, indicating a high internal reliability of each trajectory. Next, although two of the three trajectory groups were relatively large, Group 1 was very small (only about 4% of participants). Future studies should include a larger number of participants to determine if this trajectory exists across other samples, and to investigate factors that may influence membership in each group. Despite its limitations, there are considerable strengths to this study. We used a longitudinal design with repeat measures to objectively measure physical activity among a diverse sample of American children as they transitioned into adolescence. Group-based trajectory analysis, an emerging statistical methodology in the field, was applied to further explore the decline and identify subgroups of children who follow distinct physical activity trajectories. Two trajectory groups fit the typical decline of physical activity, but one trajectory group maintained and then increased in physical activity over time. Additional work is needed to better understand the characteristics of children in each trajectory group, which could facilitate public health efforts to attenuate the decline in physical activity from childhood to adolescence.

## Conclusions

Objectively-measured physical activity was monitored in a cohort of U.S. children as they transitioned from elementary to high school. Change in physical activity was assessed with group-based trajectory analysis, and three distinct patterns were identified. Two of the groups demonstrated gradual age-related decreases in physical activity, with one of the two groups observed to be consistently more active than the other. The third group was quite distinct in that it maintained a relatively high level of physical activity across the observed age range. We conclude that some children, albeit a minority, avoid the typical age-related decrease in physical activity. We recommend that future studies be undertaken to identify the factors that distinguish this group and that could be used in designing physical activity promotion programs for youth.
